# Physics Models of Plasmonics: Single Nanoparticle, Complex Single Nanoparticle, Nanodimer, and Single Nanoparticle over Metallic Thin Film

**DOI:** 10.1007/s11468-017-0598-x

**Published:** 2017-05-24

**Authors:** Wenbing Li

**Affiliations:** Institute for Frontier Materials, Deakin University, Locked Bag 20000, Geelong, VIC 3220 Australia

**Keywords:** Classical electromagnetics model, Quantum model, Plasmonics, Nanoparticle, Nanodimer

## Abstract

The physics models of plasmonics for single nanoparticle, complex single nanoparticle, nanodimer, and single nanoparticle over a metallic thin film with an isolation layer, have been reviewed in this article. In nanoscale, the localized plasmonics from the single nanoparticle, hybrid single nanoparticle, and nanodimer, can be illustrated by classical electrodynamics. When the space of a nanodimer downs to subnanometer, the classical electrodynamics would fail to predict the resonance spectrum or dispersion of the nanostructures. The quantum model and quantum-corrected electrodynamics model, are introduced to deal with this problem. For the single nanoparticle over a metallic thin film with an isolation layer, the plasmonic resonance and the enhanced local field depend on the thickness of the isolation layer strongly. When the isolation layer thickness goes down to subnanometer, the classical electromagnetics model would be replaced by the quantum model for illustrating of the plasmonics. The physics models of plasmonics have wide applications in design and fabrication of the metallic nanostructure for further research.

## Introduction

Plasmonics has recently attracted extensive interest in applications in photonics [1] such as waveguides [1], optic circuits [2, 3], lasers [4], photodetectors [4], modulators [5–7], biosensors [8, 9], and bio-images [10], and the manipulation of photons in subwavelength by designing unique nanostructures has in particularly generated enormous new opportunities [1, 7, 8]. One of the most attractive applications is in biology, where plasmonics substrate can be used as surface-enhanced Raman scattering biosensor to detect DNA, adapter, protein, enzyme, peptide, cancer cells, and antibody [11], and even single molecule [11, 12].

The interaction between light and noble metallic nanostructures determines the optics phenomena, and therefore the design of appropriate nanostructures is critical for specific applications in different areas. In the subwavelength region, the propagation plasmonics in planar metallic thin film and the interaction of light wave with single noble metallic particle were firstly developed, and the frequency-dependent plasmon resonance was then appeared in the scattering area [13]. The high sensitivity of dielectric constant of the plasmon resonance for noble nanoparticles to environment variation can result in successful biological measurement based on refractive index [4, 14]. The enhanced-near-field close to the nanoparticles could enhance the radiation from quantum wells [11, 12, 15], quantum dots [16], fluorescence [17], and molecules [10, 14, 18] with applications in photonics [5], biophotonics [14, 19], bioimage [20], and Raman scattering [10, 14, 18, 21].

Plasmonics from a single nanoparticle of complex shapes [22–25] and closely spaced nanoparticles such as dimer [24, 26], can split plasmon resonance as bonding mode and anti-bonding mode, similar to the orbital theory of molecules with a special attention to the junction between nanoparticles [26–32]. The plasmon resonance shift and electric field distribution in the junction depend on interception distance in nanoscale which can be explained using classical electrodynamics [26, 27] in subwavelength regime and can be applied in surface-enhanced Raman scattering and biosensors [14, 33]. With the decrease of the interception distance in nanoscale, the electric field in the junction would enhance, and the bonding plasmon would redshift, as predicted by the classical electromagnetic theory [26, 27]. But when the size goes to nanoscale or atomic scale, the classical electromagnetic theory would fail to explain the spectrum of the closely spaced dimers, which need to get support from the quantum theory [29–32]. In this case, the electrons can tunnel from one gold nanoparticle to another since the atomic scale distances between the two nanoparticles in the dimer, while the electrons interact with the incident light wave. For the classical electromagnetic theory, the tunneling of the electrons is not in consideration. A modification of the classical electromagnetic theory can also give a good explain of the spectrum of the closely spaced nanodimer [30]. The special resonance spectrum of the gold nanodimer can be seen as the coupling between the localized plasmonics from single nanoparticle. With a big interception between two nanoparticles as larger than the critical gap [31] (0.31 nm by Savage [31]), it can be explained with the classical electromagnetics theory; while the interception narrows to less than the critical gap, the classical electromagnetic theory would fail, and people need to use the quantum theory to explain it.

The same thing happens for the coupling between the localized-surface plasma and the surface-propagating plasma, which is defined as a gold nanoparticle closely over a gold thin film [34]. When the space is above atomic scale, it can be explained by classical electromagnetic theory [34], but while it goes down to subnanoscale, it can only be explained by quantum theory [35]. In this review, the physics models based on single nanoparticle, complex single nanoparticle, dimer, and single nanoparticle over a thin metallic film, would be summarized for the future design and understanding of related nanofabrication and applications research.

## Surface Plasmonics of Single Nanoparticle

Surface plasma describes the interaction between light and matter, especially, the interaction between light and metal material. It is a kind of wave that propagate along the interface of dielectric material and metal material caused by the function of free electrons in metal and the electric field from the incident light [36]. The phenomena can be illustrated by classical electromagnetic theory, namely, the solution of Maxwell’s equations [36]. On one side, the surface-propagating plasmon propagates along the interface between metal and dielectric. On the other side, in the vertical direction, the field named as evanescent field, would decrease exponentially both in the dielectric area and the metal area. Actually, in mathematics, the electric potential at the interface can be expressed simply as [36],

1 $$ \phi ( z)\propto {e}^{i\beta x}{e}^{\pm {k}_{1,2} z} $$

Since the electric field is the grade of the electric potential, the decay of the evanescent field in metal and dielectric area can be also seen in expression (1). The propagation constant is determined by [36]

2 $$ \beta ={k}_0\sqrt{\frac{\varepsilon_1{\varepsilon}_2}{\varepsilon_1+{\varepsilon}_2}} $$

It is a constant that to characterize the signature of the propagation for the surface propagation plasma, also named as dispersion relationship [36]. In expression (2), *ε*_1_ and *ε*_2_ represent the dielectric function of metal and dielectric materials, respectively. The surface modes appear while the propagation constant approaches infinity, which means that the dielectric function of metal material and dielectric material satisfy the following expression [36]:

3 $$ {\varepsilon}_1\left(\omega \right)+{\varepsilon}_2=0 $$

**Fig. 1:**
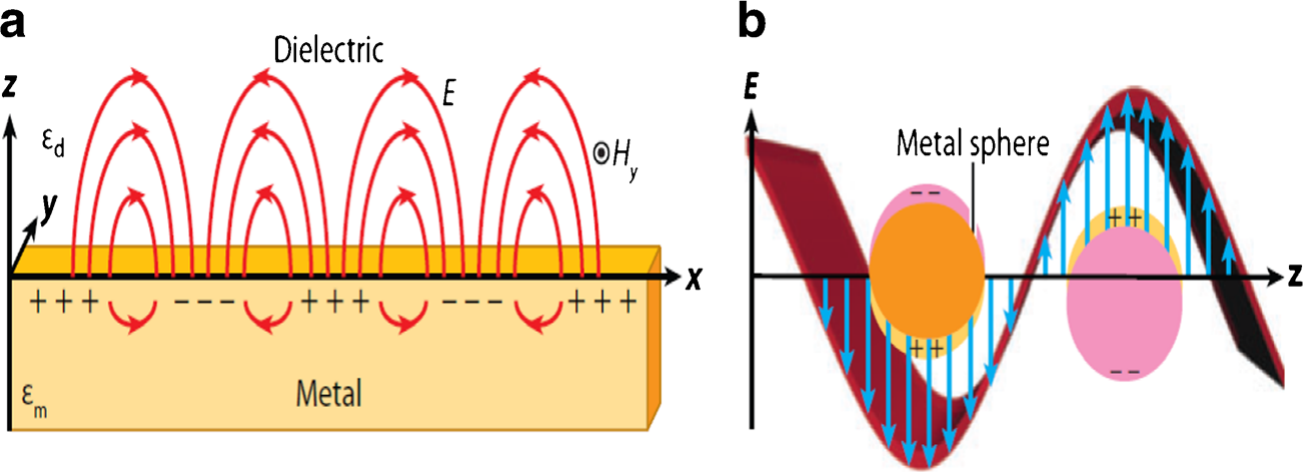
Surface propagation plasmonics (**a**) and localized surface plasmonics (**b**) [37]

While the surface propagating plasmon is a dispersive propagation excitation at the interface between a conductor and dielectric, the local surface plasma is a localized excitation corresponding to the interaction between the free electrons oscillation in the metal nanoparticles and the electric field of the incident light (Fig. 1b). Its mode comes from the scattering of a subwavelength size nanoparticles. The restoring force of the resonant electrons leads to enhanced-electric field both inside and outside of the nanoparticles.

As the nanoparticle size is in subwavelength (less than the incident wavelength), regardless of the retardation of electrons polarization, a quasistatic approximation, can be satisfied, for the Laplace equation, where the harmonically oscillation electric field of the incident light can be seen as a static field for the nanoparticle.

For a perfect nanosphere with a radius *a*, with the incident light electric field *E*_0_, the resonance peak, or LSPR (Local Surface Plasmonic Resonance) is determined by [36]

4 $$ \operatorname{Re}\left(\varepsilon \right)=-2{\varepsilon}_m $$

In this condition, the strongest extinction will be achieved, thus, it is key for the enhancement of electric field with the nanoparticles. Noble metal is a kind of best plasmonics materials in this sense. Beside metal materials, some semiconductors like ZnO [38, 39], and also graphene [40, 41] have some weak plasmonics effect, which leads to some related application. The dielectric function of metal depends on frequency or wavelength of the incident light, which results in the different color variation of noble metal nanoparticles with different angle incident of light. Variation of the media dielectric via changing of the media material would lead to the shift of the peak of extinction curve. This principle has led to use the LSPR as refractive index sensor in biosensing [14, 42]. The shift of the resonance peak of LSPR can be determined by [14]

5 $$ \varDelta {\lambda}_{\max }= m\Delta n\left[1- \exp \left(-2 d/{l}_d\right)\right] $$

where *m* is the bulk refractive index response of the nanoparticles, *Δn* is the refractive index change of the adsorbate on the nanoparticles, *d* is the absorbate layer thickness, and *l*_*d*_ is the characteristic electromagnetic field decay length.

Actually, the quasistatic approximation solution is a simple special case and has its limitation in application (but enough to clarify the physics behind LSPR) [43]. The key is that the size of the nanosphere must be smaller than the wavelength, in which the field in the light wave can be seen as a quasistatic field to simplify the model [26]. Otherwise, the plasmonics resonance would be size and shape dependent for the retardation effect [44–46]. For large nanoparticles whose size is comparable with the incident wavelength or larger than the wavelength, retardation effect has a huge influence, and the quasistatic approximation would fail to predict the extinction section and polarizability. In this condition, the dipolar plasmon peak redshifts to long wavelength and the peak broadens, meanwhile higher order modes appear at high energy in the extinction curve [47]. By contrast, for the small nanoparticles with a diameter less than 10 nm, the dimension of the nanoparticle is smaller than the mean free path of the free electron gas [36]. In other words, the electrons are confined in the 3D nanoparticle domain, which needs the help of quantum theory [48, 49]. In this condition, the local plasma excitation can produce radiation emission, or thermal emission, which leads to the application in light emission devices as OLED [50] and biomedicine [51].

**Fig. 2:**
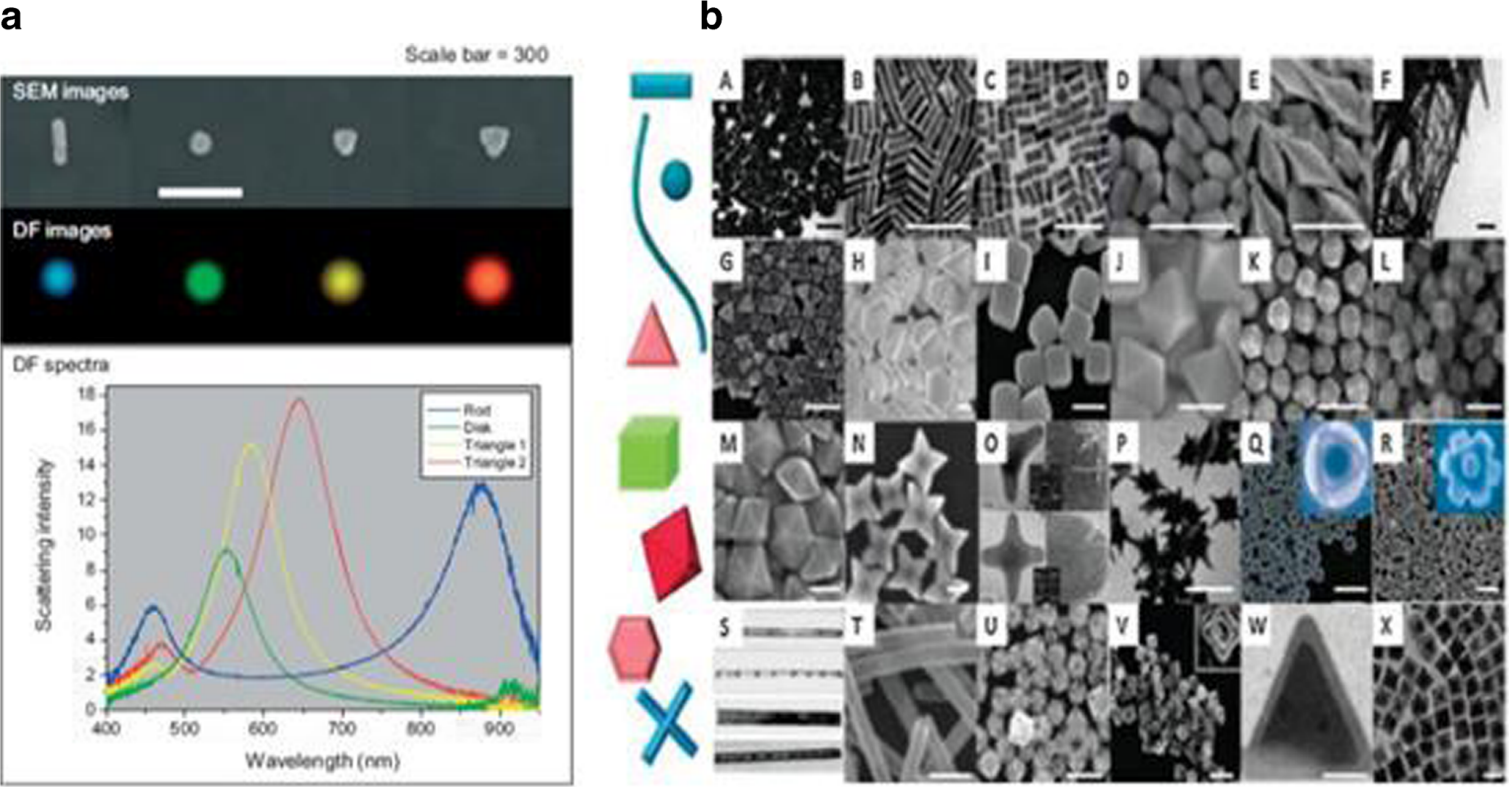
**a** LSPR resonance depends on particle shape [45]. **b** Noble metal nanoparticles with different shapes by chemical synthesis [46, 52, 54]

In order to get a high enhancement of the electric field, a high eccentricity of the nanoparticles is necessary for nanostructures design and process. The SEM and DF images of some Electrons Beams Lithography (EBL) which fabricated metallic nanoparticles, such as nanorod, nanodisk, and nanotriangles have been shown in Fig. 2a [45].Though these nanoparticles have the same height as 20 nm, and sit on the same substrate such as the ITO (20 nm)-coated silica substrate, there is huge difference in resonance peak for each nanoparticle with different shape. Besides EBL, the shape-controlled chemistry synthesis makes different kinds of noble metallic nanoparticles possible to meet the physics [46, 52], which opens a fantastic paradise of the plasmonic optics for nanoparticles and leads to wide applications.

The resonance of plasmonics has led to many different kinds of applications in photonics. For example, embedding the nanoparticles in solar cells would increase the optic path length of photons and improve the energy transfer efficiency much [53]. Rather than using in photovoltaics, Okamoto [11, 12] discovered that the coupling between Ag surface plasmonics and InGaN quantum wells would lead to a third energy-transfer channel of electron-hole pairs rather than radiation recombination and nonradiation recombination. Since the lifetime of plasmonics is shorter than the nonradiation recombination electron-hole pairs, the energy transferred to the plasmonics can be re-emitted as photons, thus, it has been reported as an effective way to improve the internal quantum efficiency of light-emission devices [11, 12] .

## Hybrid Plasmonics of Complex Single Nanoparticles

The propagating surface plasmon polariton at the interface between conductor and dielectric, and the localized-surface plasmon have been discussed above. However, the plasmonics from different metal nanoparticles, conductor, or even from different part of a single complex nanostructure would couple with each other, resulting in variations in the frequency and resonance. Therefore, a hybridization model is needed to explain the optics. Nanoshell [22], nanomatryushka [23], and nanorice [25] are the most attractive single hybrid plasmonics nanostructures.

**Fig. 3:**
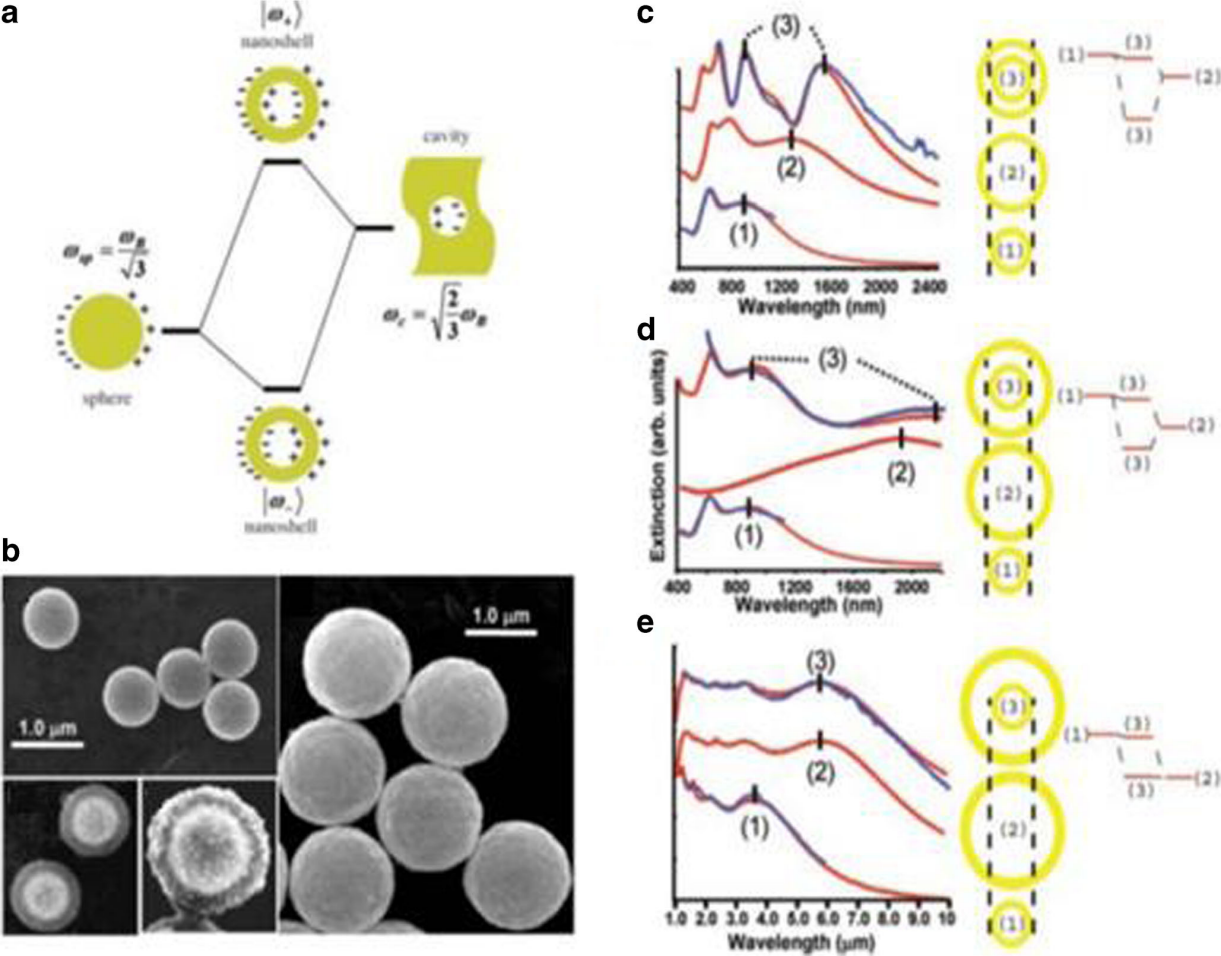
Hybridization of plasmonics of concentric nanoshell. **a** Energy diagram illustrates the hybridization of plasmon from sphere and cavity. **b** SEM images of the concentric nanoshells. **c** Concentric nanoshell with a dimension of *a*_1_/*b*_1_/*a*_2_/*b*_2_ = *a*_1_/*b*_1_/*a*_2_/*b*_2_ = 80 nm/107 nm/135 nm/157 nm. **d** cConcentric nanoshell with a dimension of *a*_1_/*b*_1_/*a*_2_/*b*_2_ = 77 nm/102 nm/141 nm/145 nm. **e** Concentric nanoshell with a dimension of *a*_1_/*b*_1_/*a*_2_/*b*_2_ = 396 nm/418 nm/654 nm/693 nm [22]

For a special nanoshell with a cavity in the metal structure, the plasmonic’s response is highly dependent on the geometry. Actually, it can be seen as a kind of interaction of the plasmonics from a nanosphere and the plasmonics from a cavity (See the schematic of nanosphere and nanocavity-combined concentric nanoshell in Fig. 3a. The plasmonics from the nanosphere and the nanocavity excite surface charges at the inner and outer surface of the metal shell. The interaction is dependent on the thickness of the nanoshell strongly. Furthermore, it leads to the splitting of the plasmonics resonance and produces two new resonances: the symmetrically coupled or bonding plasmon ∣*ω*_−_>, and the anti-symmetrically coupled or anti-bonding plasmon ∣*ω*_+_>, while angular number *l* > 1. The splitting resonance frequency can be expressed as [22, 55]

6 $$ {\omega_{l\pm}}^2=\frac{{\omega_B}^2}{2}\left[1\pm \frac{1}{2 l+1}\sqrt{1+4 l\left( l+1\right){\left(\frac{a}{b}\right)}^{2 l+1}}\right] $$

where *ω*_*B*_ is the bulk plasmonics of the metal, which is determined by the density of the free electrons and the effective mass of electrons*ω*_*B*_ = (4*πe* ^2^ *n*_0_/*m*_*e*_)^1/2^; *a* and *b* are the external and internal radius of the nanoshell, respectively. The derivation of the hybridization plasmonics of the nanoshell involves in the solution of continuity equation and Laplace equation with the incompressible fluid assumption, which could be predicted by the classical Mie theory [56]. Furthermore, multilayers of concentric metallic nanoshells have also been investigated with the incompressible fluid model by Prodan [56]. Rather than the single layer metallic nanoshell, the plasmonic resonance of multilayers metallic nanoshell is very complex. A multilayer concentric metallic nanoshell with layer number of *N*, inner radius *a*_*j*,_ and outer radius *b*_*j*_ can be gotten by solving the Lagrange equation and given by the following Eigen equation: [57]

7 $$ \det \left[{\omega}^2\hat{T}-\frac{1}{2}\left(\hat{V}+{\hat{V}}^T\right)\right]=0 $$

The matrix $\widehat{T}$ in the Eigen equation is a block diagonal matrix determined by kinetic energy [57]:

8 $$ \widehat{T}=\left(\begin{array}{ccc}\hfill {\hat{T}}_1\hfill & \hfill \hfill & \hfill 0\hfill \\ {}\hfill \hfill & \hfill \dots \hfill & \hfill \hfill \\ {}\hfill 0\hfill & \hfill \hfill & \hfill {\hat{T}}_N\hfill \end{array}\right) $$

9 $$ {\hat{T}}_i=\left(1-{x}_i^{2 l+1}\right)\left(\begin{array}{cc}\hfill \left( l+1\right){a}_i^{-2 l-1}\hfill & \hfill 0\hfill \\ {}\hfill 0\hfill & \hfill {lb}_i^{2 l+1}\hfill \end{array}\right) $$

The matrix $\widehat{V}$ is the electrostatic energy determined by the surface conduction electrons density *σ*_*ci*_, *σ*_*si*,_ and the electric potential ${\phi}_{lm}^j\left({a}_i\right)$, ${\phi}_{lm}^j\left({b}_i\right)$ [57].

10 $$ {V}_{lm}={\sum}_{i, j}\left[{a}_i^2{\sigma}_{ci}{\phi}_{lm}^j\left({a}_i\right)+{b}_i^2{\sigma}_{si}{\phi}_{lm}^j\left({b}_i\right)\right] $$

A two-layer concentric metallic nanoshell with a silica core and a gold shell has been synthesized with Stober method (Fig. 3b) [22], and the experimental (blue) and theoretical extinction curves (red) [22] have been given in Fig. 3c–d to illustrate the hybridization concept of plasmonics. The hybridization plasmonics can be seen as the combination of two individual metallic nanoshells: nanoshell 1 + nanoshell 2. The interception between the two nanoshells determined how strongly the individual plasmon coupled with each other. In Fig. 3c, for the concentric nanoshell with a radius distribution of *a*_1_/*b*_1_/*a*_2_/*b*_2_ = 80 nm/107 nm/135 nm/157 nm, there is a very small interception of 28 nm between the inner nanoshell and the outer nanoshell. There is a very strong coupling between the plasmon from the inner and the outer nanoshell because the resonance from each nanoshell was so close to each other, which leads to strongly asymmetric bonding. For comparison, a weak coupling of plasmon has been shown in Fig. 3d. Since the two concentric nanoshells with an increased interception 39 nm (*a*_1_/*b*_1_/*a*_2_/*b*_2_ = 77 nm/102 nm/141 nm/145 nm), the plasmon resonance from individual nanoshells is far from each other. Furthermore, while the interception increases to 236 nm (*a*_1_/*b*_1_/*a*_2_/*b*_2_ = 396 nm/418 nm/654 nm/693 nm) which showed in Fig.3e, there is no coupling between the plasmon from inner and outer nanoshells, or in other words, a full decoupling of plasmon appears. The SEM images in Fig. 3b have shown the nanoshells in each step of the chemistry synthesis, where the silica nanoparticles (inner core), silica-encapsulated nanoshell, silica-coated nanoshell seeded with Au-colloid to initiate the growth of the second gold layer, and the completed two-layer concentric nanoshell, respectively. The multilayer concentric nanoshell model illustrated above is based on the classical theory.

Strong quantum effect on the nanomatryushka appears while the core-shell space decreases to 0.5 nm, as observed by Vikram [23]. This fantastic phenomenon of plasmonics and its broad applications in subwavelength optics, biotechnology, and medicine have stimulated strong interests. As pioneer researchers, E. Prodan and P. Nordlaner [22, 55, 57] have tried to understand the physics of hybridization model of plasmonics.

Beside the symmetric metallic nanoshells, the open-nanoshell structures, which are asymmetrical, have also been studied theoretically [58]. The hybridization concept is still applicable to analyze such kinds of nanostructures but are analyzed as the hybrid of a perfect nanoshell plasmon and nanohole dipole plasmon [58].

**Fig. 4:**
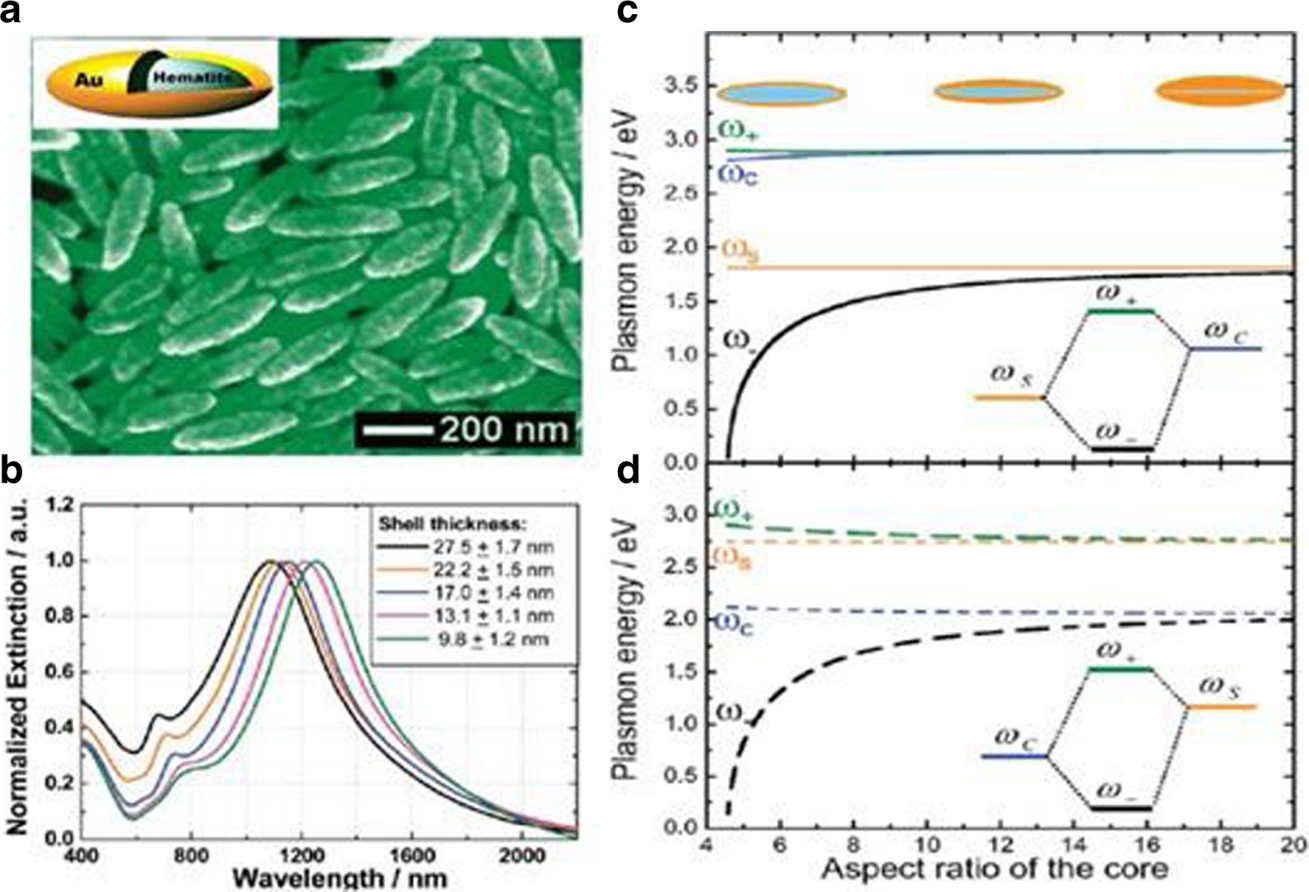
SEM image of Au nanorices with a hematite core and a gold shell (**a**) extinction spectrum depends on shell thickness, and plasmonics hybridization of nanorice (**b**), longitudinal (**c**), and transverse (**d**) plasmon energy depends on the aspect ratio [24, 25]

The nanorice combined of a dielectric core and noble metal shell is similar to the nanoshell, which can be seen as the hybridization of cavity plasmon and ellipsoid plasmon. Hui Wang et al. [24] synthesized a nanorice with a hematite core and Au shell (Fig. 4a) using chemistry method. The solution of the resonance frequency can be expressed as [25]follows:

11 $$ {\omega_{lm}}^2\left(\alpha \right)={\omega_B}^2\frac{P_{lm}\left( \cosh \alpha \right){Q}_{lm}^{\hbox{'}}\left( \cosh \alpha \right)}{\varepsilon {P}_{lm}\left( \cosh \alpha \right){Q}_{lm}\left( \cosh \alpha \right)-{\varepsilon}_m{P_{lm}}^{\hbox{'}}\left( \cosh \alpha \right){Q}_{lm}\left( \cosh \alpha \right)} $$

where *α* is a constant of the elliptical spheroid (cosh*α* is the aspect ratio of the spheroid), *P*_*lm*_ and *Q*_*lm*_ are the associated Legendre polynomials of the first and the second kind respectively, *ε* and *ε*_*m*_ are the dielectric of the metal and the media core, respectively. The resonance of the nanorice depends on the shell thickness strongly, and it redshifts with the decrease of the thickness (Fig. 4b). A strong near-field forms close to the two sharp tips of the nanorice, which leads to high sensitivity of the surface plasmon resonance nanosensor. With a decrease in shell thickness, the sensitivity further enhances because of the improved coupling between the plasmons from inner and outer of the nanorice [26]. Many molecular probes, such as carbon disulfide, toluene, n-hexane, and methonal, have been tested, and high sensitivity has been achieved with this nanorice. The numerical simulation of the nanorice based on eq. (12) are shown in Fig. 4c, d. The plasmon energy of the cavity and the spheriod depends on the aspect ratio, which results in the hybridization of plasmonics, either for longitudinal polarization or transverse polarization. When the aspect ratio decreases, the hybridization between cavity and spheroid couple with each other strongly, leading to the big splitting of bonding plasmon and anti-bonding plasmon.

## Classical Hybrid Plasmon of Nanodimer

**Fig. 5:**
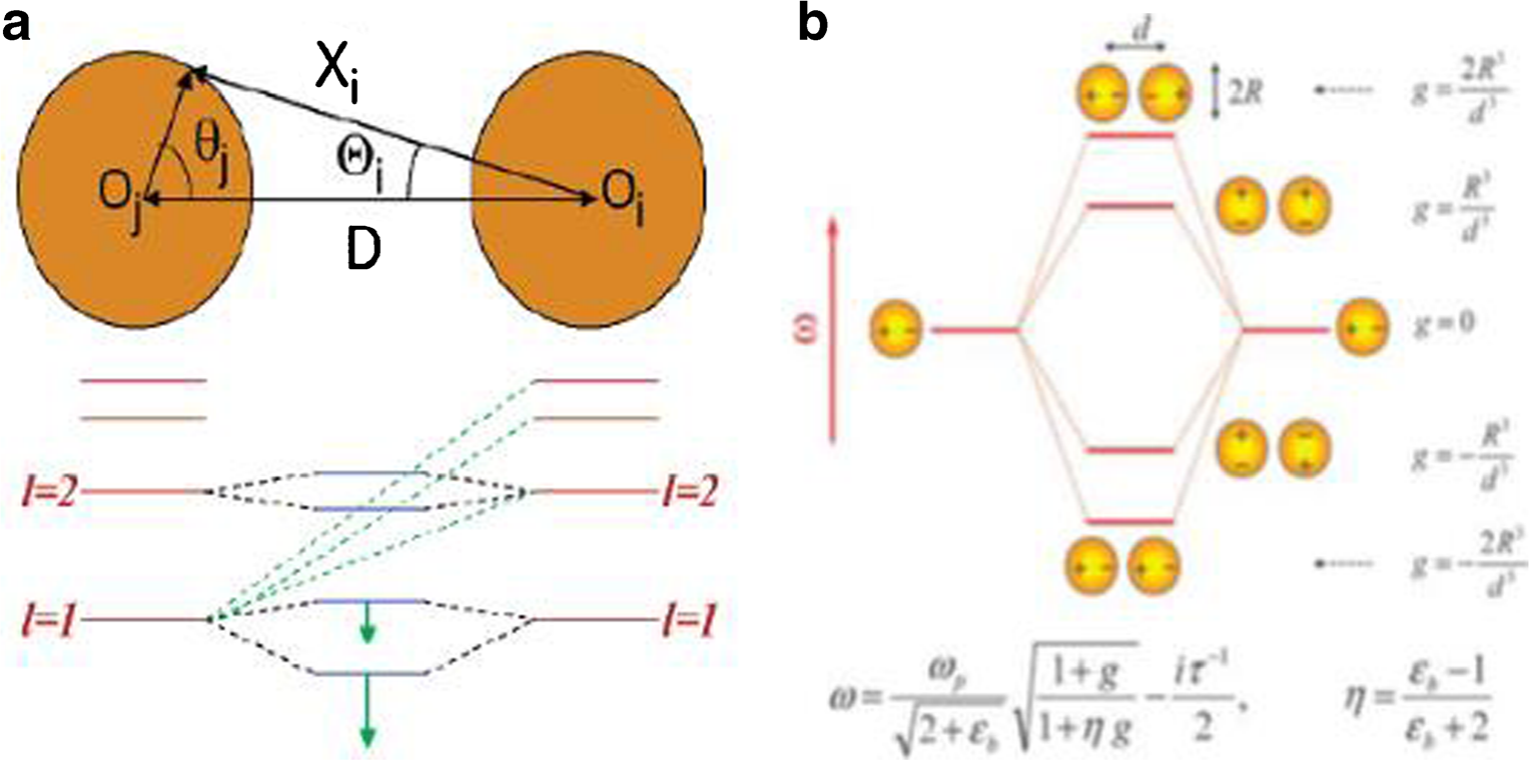
Plasmon hybridization in interacting nanoparticles. (**a**) Schematic diagram of a nanospheres dimer [26]. **b** Energy level of nanospheres dimer [27]

The hybrid model of the plasmonic has been described for the single complex nanoparticle above. It also happens to two closely spaced nanoparticles, namely, nanodimer. The hybrid model has a nickname as “artificial molecule” for the splitting of plasmon resonance from individual. The problem becomes quite complex with electromagnetic theory. Nordlander [26] developed the theory based on an assumption that the electron gas in the nanoparticles can be seen as a kind of charged, incompressible, and nonrotational liquid over the head of a rigid. In Fig. 5a, with Nordlander’s theory [26], the plasmon resonance can be derived from Euler-Lagrange equation by solving an Eigen equation as

12 $$ \det \left[{A}_{ij}^{(m)}-{\omega}^2\right]=0 $$

If the size of the dimer can be much smaller than the incident wavelength, the quasistatic assumption can be used and the retardation effect can be neglected. The matrix “A” in the Eigen equation is expressed as [26]

13 $$ {A}_{i j}^{(m)}={\omega_i}^2{\delta}_{i j}+\frac{{\omega_B}^2}{8\pi}\left({V}_{i j}^{(m)}+{V}_{ji}^{(m)}\right) $$

where *l*_*i*_ and *l*_*j*_ are the angular momentum of *i* and *j* mode of the plasmon, respectively, and *δ*_*ij*_ is the surface charge density. The Coulomb potential between two nanoparticles is expressed as [26]

14 $$ {V}_{i j}^{(m)}(D)=4\pi \sqrt{l_i{l}_j{R}_i^{2{l}_i+1}{R}_j}\int d{\theta}_j \sin {\theta}_j\frac{P_{l_i}^m\left( \cos {\Theta}_i\left({\theta}_j\right)\right)}{\left(2{l}_i+1\right){X}_i{\left({\theta}_j\right)}^{l_i+1}}{P}_{l_j}^m\left( \cos \left({\theta}_j\right)\right) $$

where *R* is the radius of the nanoparticle and *D* is the distance between two nanoparticles. According to Norlander’s research [26], if the interception between the two nanoparticles is large, the interaction between each nanoparticle is weak, and the essential bonding and anti-bonding would have the same angular moment.

If the size of the dimer is large, and the Coulomb interaction is not available, which needs to replace it with Van Der Vaal’s interaction [27],

15 $$ {E}_{VDW}=\frac{\hslash }{2}\sum_j\left({\omega}_j-{\omega}_j^0\right) $$

where *ω*_*j*_ and ${\omega}_j^0$ represent the interaction and non-interaction particles’ mode frequency. If the interception is larger than the radius of the nanoparticle, the dominant modes would appear, in which the Van Der Vaal’s function is expressed as [27]

16 $$ {E}_{VDW}=\frac{3\hslash {\omega}_p}{4\sqrt{2+{\omega}_b}}\left(3\eta +1\right)\left(\eta -1\right){\left(\frac{R}{d}\right)}^6 $$

where $\eta =\frac{\varepsilon_b-1}{\varepsilon_b+2}$. The splitting of the plasmon at different modes is shown in Fig. 5b.

The Discrete Dipole Approximation (DDA) is also a powerful theoretic method to investigate the optics’ properties of nanoparticles and dimers. Hao and Schatz [59] investigated the influence of dimer separation and orientation with DDA method, and claimed that the maximum electric field intensity (${\left|\overset{\rightharpoonup }{E}\right|}^2$) of dimer was about tenfold larger than that of a single nanoparticle. Based on Hao and Schatz’s theoretic investigation [59], the interception of a dimer was a key factor rather than the geometry, to achieve a high electric field in the junction, and the biggest ${\left|\overset{\rightharpoonup }{E}\right|}^2$ value was about 10^5^ with a space of 2 nm for Ag dimer. The enhancement of electric field in the dimer junction will improve the Raman intensity, which is also named as “hotspot” [36, 60, 61].

Experiment work on investigating the influence of the gap for the hybrid plasmon resonance has been undertaken by Yang, et al. [28]. The silver nanoparticle dimer was synthesized by a rational DNA-programmed self-assembly process. According to Yang et al. [28], the hybrid plasmon resonance would redshift with the decrease of the separation between the two nanoparticles until the centre-to-centre distance of the two nanoparticles equal to its diameter. Furthermore, an experiential expression could be extracted out from the experiment data as [28]

17 $$ {E}_{res}^{fit}=\left(2.77-457.36 \exp \left(-\frac{L}{Dk}\right)\right) eV $$

where *L* is the centre-to-centre distance between two nanoparticles, *D* is the diameter of the nanoparticle, and *k* is a constant co-efficiency (*k* = 0.16 ± 0.03). But for an even shorter distance between the two nanoparticles, the plasmon resonance energy became broadly distributed, and there was no regular connection between the plasmon resonance energy and the interception.

## Quantum Theory for Plasmonics of Dimer

**Fig. 6:**
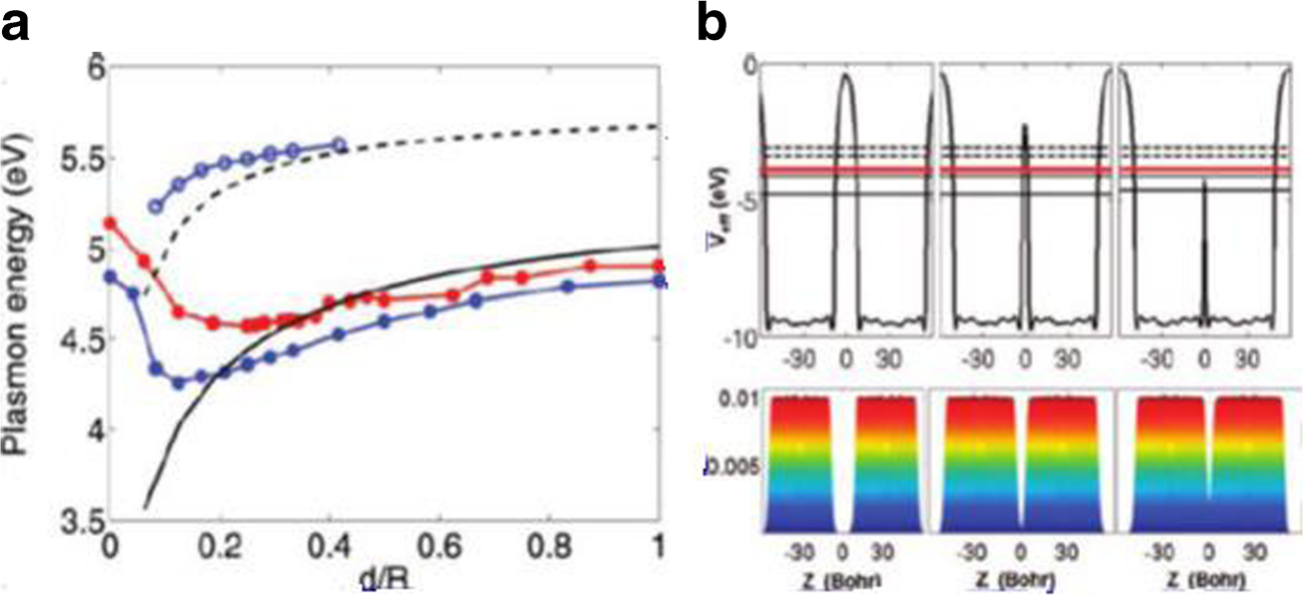
**a** Plasmon energy depends on the interception of the nanospheres. **b** Barrier potential between the nanospheres depends on the interception between the two nanospheres [29]

When the dimer space decreases, the classical theory based on electromagnetics would fail to predict the optics, and the quantum theory is required [29]. Zuloaga et al. [29] investigated the plasmon resonance of a nanoparticle dimer with full quantum theory, and the nanospheres dimer was processed with the time-dependent local density approximation (TDLDA). When the dimer space was below 1 nm, quantum effect, such as electrons tunneling through the junction and screening, would influence the plasmon resonance and enhance electric field significantly. Actually, the quantum effect led to a drastic reduction of the electric field in the junction compared with the prediction of the classical electromagnetic theory. In the region where the ratio of the edge interception between two nanospheres and the nanosphere radius satisfy 0.5 < *d*/*R* < 1 (Fig.6a), the “plasmon energy~nanospheres interception” relationship is the same for both hybrid plasmon model and the TDLDA (Time Dependent adiabatic local Density Approximation) model. But while *d*/*R* is less than 0.4, the TDLDA shows that the plasmon energy would increase (blueshift) rather than redshift predicted by hybrid plasmon model. That is for the dipolar (solid black line: hybrid plasmon model for dipolar, full circles red and blue line for dipolar with the radius of 16b and 24b, respectively). For the quadrupolar, the TDLDA and the hybrid plasmon models also show difference while *d*/*R* is less than 0.4. Furthermore, for the dimer with sphere radius of 24b, the potential between the two nanoparticles and the electron population in the junction have been calculated by Zuloaga and his colleagues (Fig. 6b [29]. Three regimes with different interceptions shown in Fig. 6b have different potential barriers and electrons population. While the interception*d* > 10*b*(0.53*nm*), it is in the classical regime, there is a huge barrier between the two nanospheres, which results in few electrons in the junction. The free electrons in each nanosphere cannot tunnel the junction to the other. In this case, the hybrid plasmon can be understood by the classical electrodynamics. With the decrease of the junction size, as in the crossover regime 4*b* < *d* < 10*b*(0.21*nm* < *d* < 0.53*nm*), the potential becomes narrower and lower, and there are finite electrons in the junction. The electrons in one nanoparticle can tunnel to another with the influence of outside electric field. In other words, electrons in one nanosphere can tunnel through the junction to occupy the unoccupied state of another nanosphere. In the charge tunneling regime *d* < 4*b*(*d* < 0.21*nm*), the potential is lower than the Fermi energy, and the electrons’ population in the junction is large. The electrons can go through the junction freely.

**Fig. 7:**
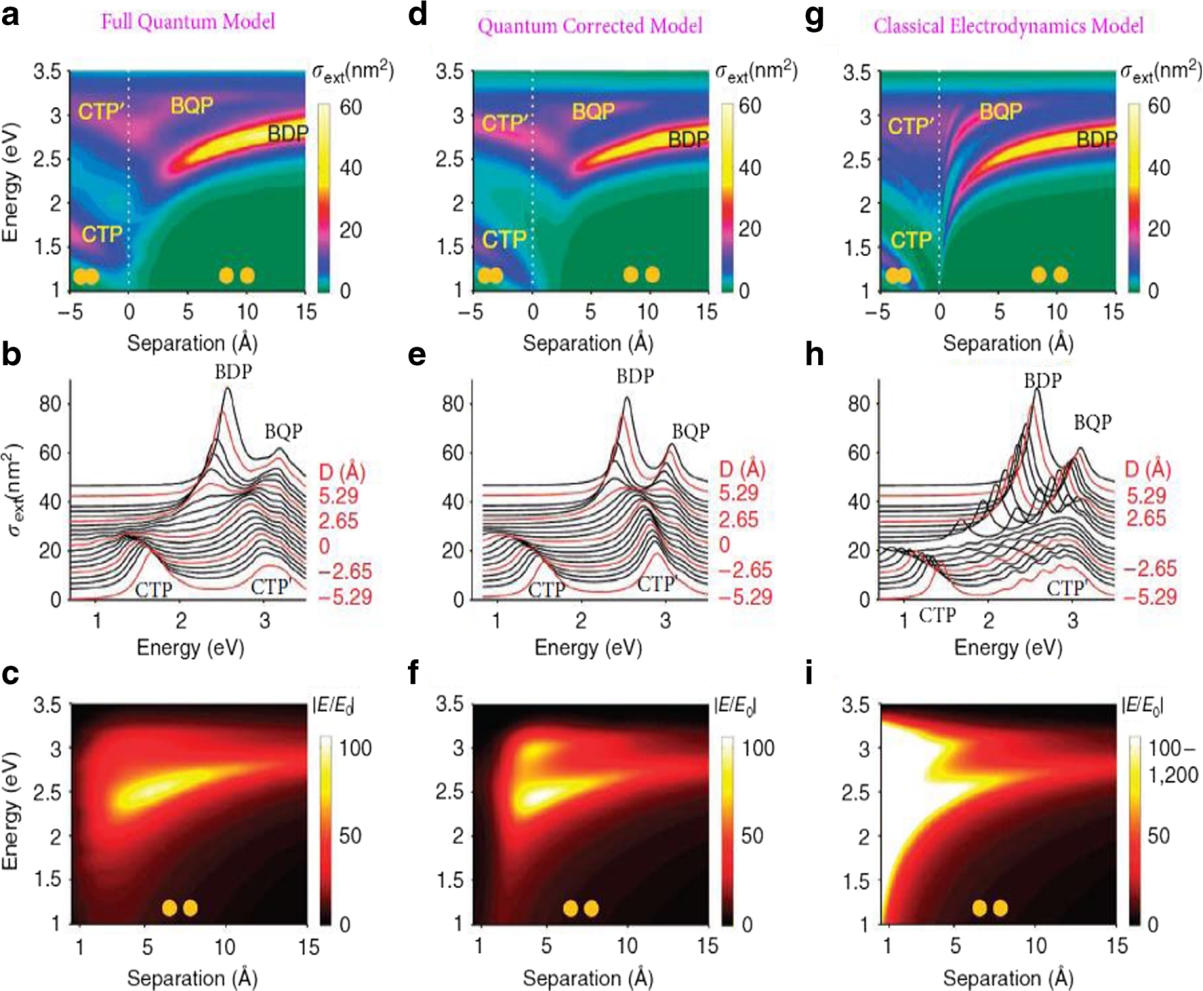
Comparison of the full quantum model (**a**, **b**, **c**), the quantum-corrected model (**d**, **e**, **f**), and the classical electrodynamics model (**g**, **h**, **i**). (**a**), (**d**), (**g**) shows the extinction spectra of the dimer as function of separation distance for the full quantum model, the quantum-corrected model, and the classical electrodynamics model, respectively. (**b**, **e**, **h**) show far field spectra from selected distance from (**a**), (**d**), and (**g**), respectively. (**c**, **f**, **i**) show local field enhancement at the centre of the junction for each dimer with an interception larger than 0.5 Å [30]

Further research on the physics model of the plasmon from dimer has revealed more details on the optics property. Esteban et al. [30] introduced the quantum-corrected model rather than the quantum theory to explain the plasmon of a dimer. For the classical electrodynamics model, the electrons density depends on the profile of the nanoparticles, there is no charge in the junction, no electrons in nanosphere can tunnel through the junction, and the conductance of the junction is zero. With the influence of the Coulomb interaction and the outside field, the electric field in the junction would increase when the interception decreased. For the full quantum model, the electrons density depends on the profile of the wave function, and the wave function of each nanosphere would overlap with a small interception. Namely, the electrons’ tunneling exists in the junction with a small interception. For the quantum-corrected model, Esteban et al. [30] assumed that there was a special dielectric in the junction connecting the two nanospheres, which can let the electrons tunnel through it, and then it can be dealt with the classical method, rather than the full quantum theory. This allows classical electrodynamics theory to predict the optics of the dimer correctly just as the full quantum theory did in super closely spaced nanospheres, where the full classical electrodynamics theory would fail. Comparison of the full quantum theory, the quantum-corrected model, and the classical electrodynamics model could help people understand the optics of the dimers (Fig. 7). The calculation results from these three models are shown in Fig. 7. (Fig. 7a–c) represents the full quantum model, (Fig. 7d–f) represents the quantum corrected model, and (Fig. 7g–i represents the classical electrodynamics model. The dimer in the calculation considered as two Na spheres of the radius *R* = 2.17*nm* in vacuum and separates by a distance symbolized as D. The incident field is a kind of planar wave, which has an electric field polarized along the dimer axis. Different resonance modes, namely, bonding dimer plasmon (BDP), bonding quadrupolar plasmon (BQP), charge transfer plasmon (CTP), and higher-order charge transfer plasmon (CTP’) have been given in Fig. 7 for the three different physics models. When the interception between the two nanospheres decreases from 1.5 to 0.5 nm, two modes, the BDP and BQP, appear in all the three models, and both of the BDP mode and BQP mode redshift. However, with further decrease of the interception from 0.5 to 0 nm (contact condition), the BDP mode would disappear in both the full quantum model and the quantum corrected model, while it still exists in the classical electrodynamics model, and continues to redshift with the decrease of the interception. The electric field in the junction in both the full quantum model and the quantum-corrected model would decrease when it reaches the maximum value as about 0.3 nm. By contrast, the electric field in the junction predicted by the classical electrodynamics model would keep increasing with the decrease of the junction interception. The decrease of the electric field in the junction predicted by the quantum theory and the quantum-corrected theory comes from the tunneling of the electrons in the junction. In the area where the two nanospheres contact with each other, the charge-transfer plasmon and higher-order charge transfer plasmon appear in all three models, and blueshift with the two nanospheres go into each other.

**Fig. 8:**
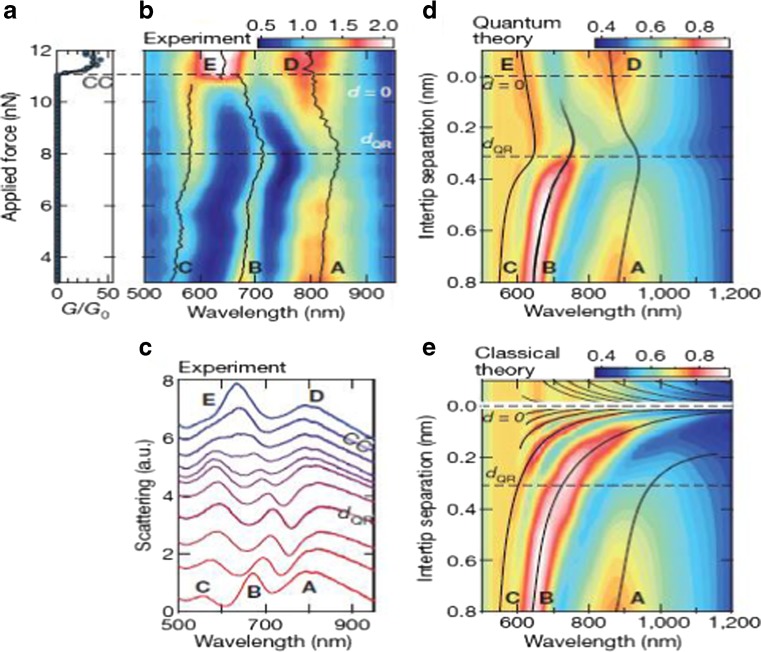
Comparison of the experiment result with quantum and classical model. Electrical conductance (**a**) and dark-field optical back-scattering (**b**) depends on applied force to the inter-tip cavity. (**c**) Selected scattering spectra from the last 1 nm to contact in (**b**). (**d**) Theoretical total scattering from a tip-tip system with quantum tunneling. (**e**) Theoretical total scattering from tip-tip system with classic calculation [31]

As the classical electrodynamics model will fail to provide correct prediction at subnanometer scale, the quantum theory should be used to investigate the hybrid plasmon. Savage et al. [31] studied the quantum region of the coupled plasmon in detail with both quantum-corrected model [30] and smart experiment with two closely spaced Au-coated AFM tips via a purpose-designed piezo platform. Compared to Esteban et al.’s pure theoretic work [30], similar results on the plasmon modes’ analysis of the experiment data, quantum theory, and quantum-corrected theory have been given by Savage et al. (Fig. 8) [31]. According to Savage et al. [31], there exists a critical gap for marking the quantum regime [31],

18 $$ {d}_{QR}= \ln \left(3 q\lambda \alpha /2\pi \right)/2 q $$

where *q* is the semi-classical electron tunneling wavenumber, *λ* is the optical plasmon wavelength, and *α* is the fine structure constant determined by the dielectric coefficient in vacuum. Based on Savage et al.’s calculation [31], while *d*_*QR*_ = 0.31*nm*, there is sufficient screening via quantum transport to overcome the increasing charge. Three interaction regimes bordered by the critical gap have been revealed.

**Fig. 9:**
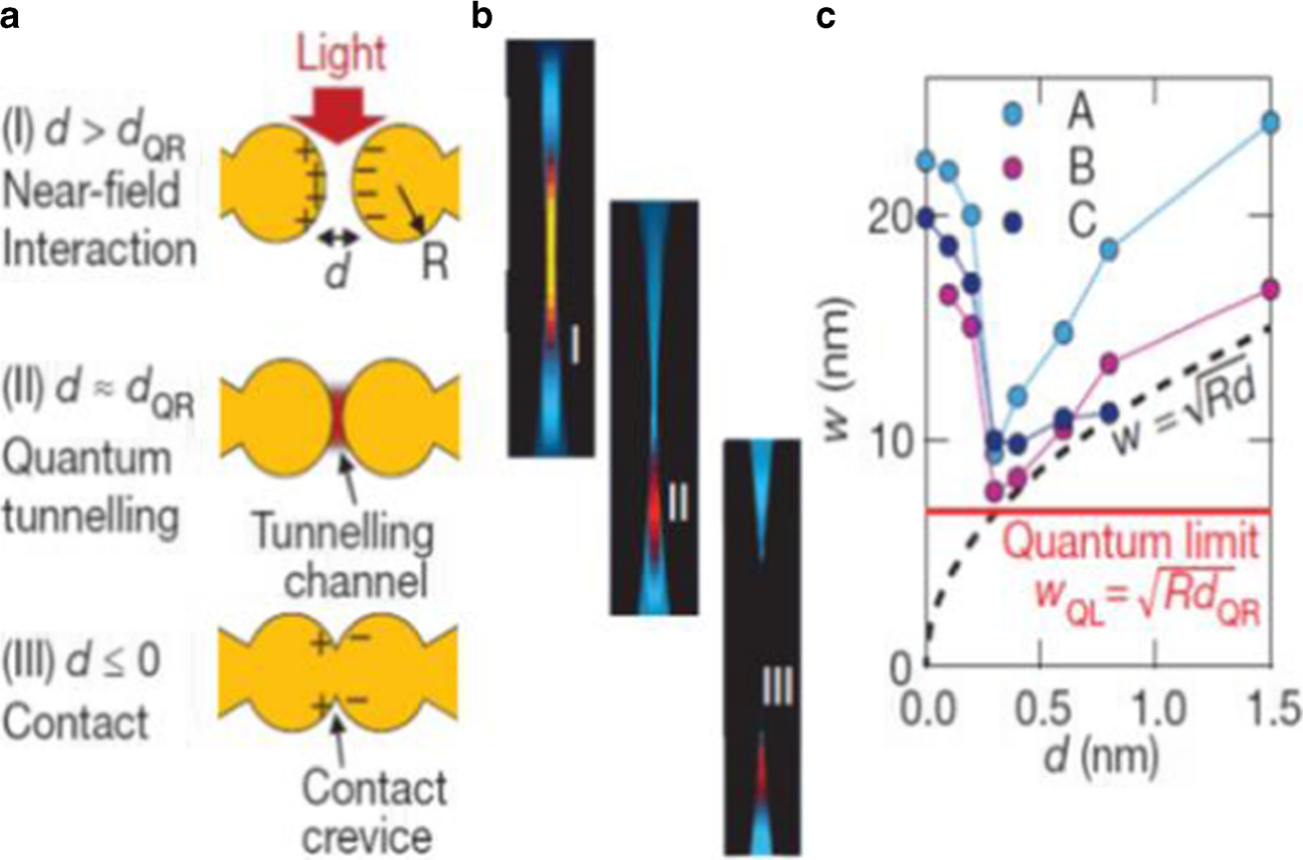
Evolution of plasmonic mode in quantum regime. (**a**) Plasmonic interactions within three regimes in experiment. (**b**) Near-field distribution for B~E mode with quantum-corrected model. (**c**) The lateral confinement width w of each mode, extracted from the simulated near-field distribution, as the cavity width decreased. *Dash line marks* the classical approximation $W=\sqrt{Rd}$. The quantum tunneling effects at *d*_*QR*_ = 0.31*nm* sets a quantum limit on mode confinement in subnanometer plasmonic cavity. [31]

(1) While *d* > *d*_*QR*_, the interaction between two nanospheres can be explained by classical electrodynamics as the near-field interaction. In this area, the plasmon resonance redshift with the decrease of the interception; (2) while 0 < *d* ≤ *d*_*QR*_, the quantum tunneling starts to function on the two nanospheres, and the classical theory will fail. With the continuing decrease of the interception, the plasmon resonance blueshift; (3) while *d* < 0, the two nanospheres connect with each other, there would be charge-transfer mode in the plasmon resonance. This has been investigated by Duan et al. [62] with connected triangle prism fabricated by Electron beam lithography, too. It would be illustrated in the following detail: In theory, actually, Liu et al. [63] has developed a model to simulate the dimer with a conductive bridge and discovered that the charge transfer would lead to a blueshift of the bonding mode and a decrease of the electric field in the junction. Savage et al. [31] also defined the lateral confinement width ${W}_{QL}=\sqrt{Rd_{QR}}$ to describe the quantum limit (Fig. 9). The nonlinear effects of the dimer with quantum theory also have been investigated recently [64].

**Fig. 10:**
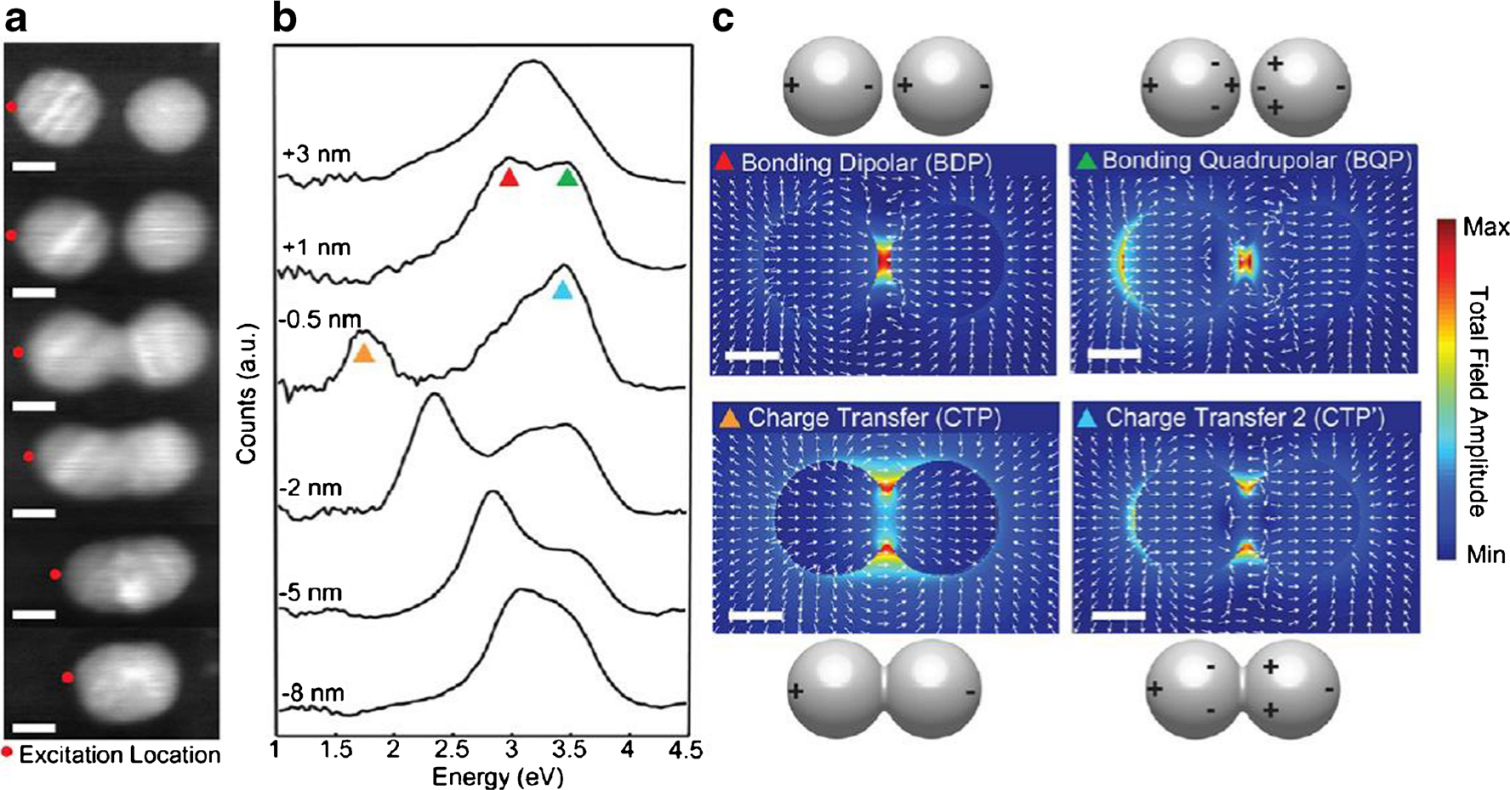
Plasmon modes analysis of dimer within quantum regime. (**a**) STM images of closely spaced-silver nanospheres by electrons beam with different interception +3 nm, +1 nm, −0.5 nm, −2 nm, −5 nm, and −8 nm. (**b**) Certain plasmonic modes with different interception between silver nanospheres from +3 nm to −8 nm. **c** Particle polarization and calculated-total field amplitude of four main contribution modes [32]

The quantum tunneling in the junction of a dimer has been verified by Scholl et al. [32] with two closely spaced silver nanoparticles. Electron beam in scanning transmission electrons microscope was used to move the 10 nm diameter silver nanospheres on a substrate, and the distance was controlled from 7 nm to 0. They could even be merged to form a single nanoparticle. Then, the electrons energy loss spectroscopy was employed to measure the plasmon modes for each dimer with different interception. The plasmon modes have been shown in Fig. 10b. With an interception of 3 nm, there was only one bonding dimer plasmon mode at 3.2 eV. When the interception decreased to 1 nm, the BDP mode redshift to 3 eV, and another mode, the bonding quadrupolar mode appeared at 3.5 eV. While the interception decreased to −0.5 nm, the charge-transfer plasmon mode appeared at 1.75 eV, and the higher-order charge transfer plasmon mode appeared at 3.45 eV. Further motion resulted in broaden of the bridge and the blueshift of the charge transfer plasmon mode. When the two silver nanoparticles were merged as a single one, a single plasmon mode appeared. The electric field for each mode in the junction was also shown in Fig. 10c. The bonding dipolar plasmon mode started to disappear when the interception decreased to 0.5 nm, because of the electrons tunneling in the junction. This could be the approval of the quantum theory and the quantum corrected theory illustrated above [30].

More works involved in using DNA as a linker between two nanoparticles have been done even at subnanometer scale. Cha et al. [65] studied the hybrid plasmon of synthesized-Au nanoparticle dimers with different interception. The 1 nm interception distance worked as a border, which divided the area into quantum region (<1 nm) and classical region (>1 nm). In the classical region, with the decrease of interception distance, the plasmon resonance redshifted; while in the quantum region, when the interception distance decreases, the plasmon resonance blueshifted to a minimum wavelength (interception = 0.8 nm), then redshifted to long wavelength. Cha et al.’s work [65] was in good agreement with the model developed by Savage et al. [31]. Similar works using DNA as the linker between Au nanoparticles have been studied by P. Busson et al. [66] and Piantanida et al. [67].

## Coupling of LSPR and SPP

Besides the coupling between metallic nanoparticles, the coupling between metallic nanoparticles and metal thin film is also quite attractive. The junction forms between the nanoparticles and a thin film via a spacer layer. The variation of the spacer thickness can change the coupling between the nanoparticle and metal thin film, which shifts the plasmon resonance and enhances the electric field, just like the interception between two nanoparticles that determined the plasmon resonance of a dimer. Actually, the metal nanoparticle over metallic thin film separated by a spacer system could also be termed as dimer, since the hybrid plasmon is seen as the nanoparticle couple with its image in the metal mirror [68].

Leveque and Martin [34] studied the coupling of LSPR and SPP via numerical method, and found that the plasmon resonance would blueshift with the increase of distance between nanoparticles and metal thin film. Mock et al. [69] studied the plasmon resonance of LSPR-SPP system with gold nanoparticles (size 60 nm in diameter) and Au thin film (thickness 45 nm) intercepted by different thickness of polyelectrolyte spacer. The dark field illumination showed that the variation of the distance between Au nanoparticles and Au thin film would influence the polarization, which resulted in a doughnut-shaped point spread at short distance. Meanwhile, the plasmon resonance would blueshift with the increase of spacer thickness. The total internal reflection (TIR) with a triangle prism showed the same result of the nanoparticle image as dark field measurement, but a little different plasmon resonance and spacer thickness relationship. The plasmon resonance blueshift first with the increase of spacer thickness, then redshift with further increase of spacer thickness. According to Mock et al.’s further research [69], for the dark field measurement, the LSPR controlled the scattering spectrum for it would preferentially excite rather than the SPP in Au thin film. But for the total internal reflection measurement, the LSPR controlled the scattering spectrum at a thin spacer, while the SPP controlled the scattering spectrum at a thick spacer. Anyway, both of the dark field measurement and the total internal reflection suggested that the plasmon resonance was sensitive to the space thickness.

**Fig. 11:**
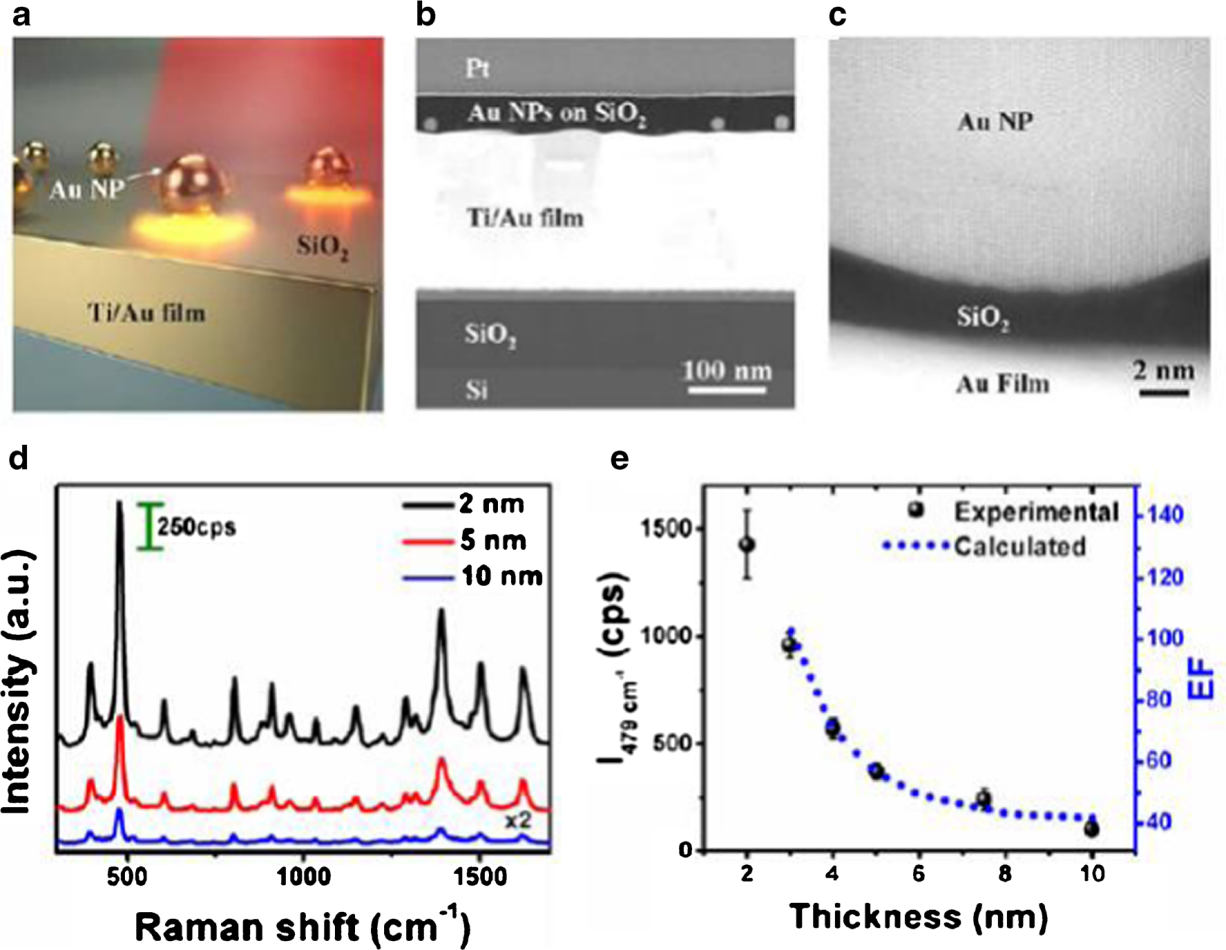
Plasmon property of the Au nanoparticle over Au thin film with ultrathin SiO_2_ spacer. (**a**) Schematic of the Au NP/SiO_2_/Au. (**b**) STEM image of the cross section for the multilayers structure. (**c**) A high resolution TEM image of Au NP/SiO_2_/Au thin film. (**d**) Raman spectra of APTES on the Au NP/SiO_2_/Au thin film with different SiO_2_ thickness.(**e**) Raman shift (479 cm^−1^) intensity depends on the SiO_2_ thickness. [70]

Rather than using polyelectrolyte as spacer, Mubeen et al. [70] investigated the plasmon properties of gold nanoparticles over a gold mirror separated by a thin SiO_2_ film deposited with atomic layer deposition (ALD). The model is shown schematically in Fig. 11a, STEM image of the cross section is shown in Fig. 11b, and a high resolution TEM image is shown in Fig. 11c. The nanostructure, Au nanoparticle over Au film separated by an ultrathin SiO_2_ spacer could be seen in Fig. 11c clearly. The surface-enhanced Raman microscopy characterization was used as a probe for inspecting the plasmon properties of this model. Thionine was used as the molecule probe. The Raman intensity showed that it depended on the spacer thickness strongly. With the decrease of the spacer thickness, the intensity of 479/cm shift would improve. The simulation result showed the enhancement factor would improve from 40 to 140, with the decrease of SiO_2_ spacer of 10 to 2 nm. That is an indirect verification of the electric field enhancement from the coupling of LSPR and SPP. Just as the plasmon in nanoparticles dimer, the electric field would improve much in junction with the decrease of the interception. Further investigation found that the Raman intensity also strongly depended on the angle of incident excitation light and would reach its maximum at 60 °

**Fig. 12:**
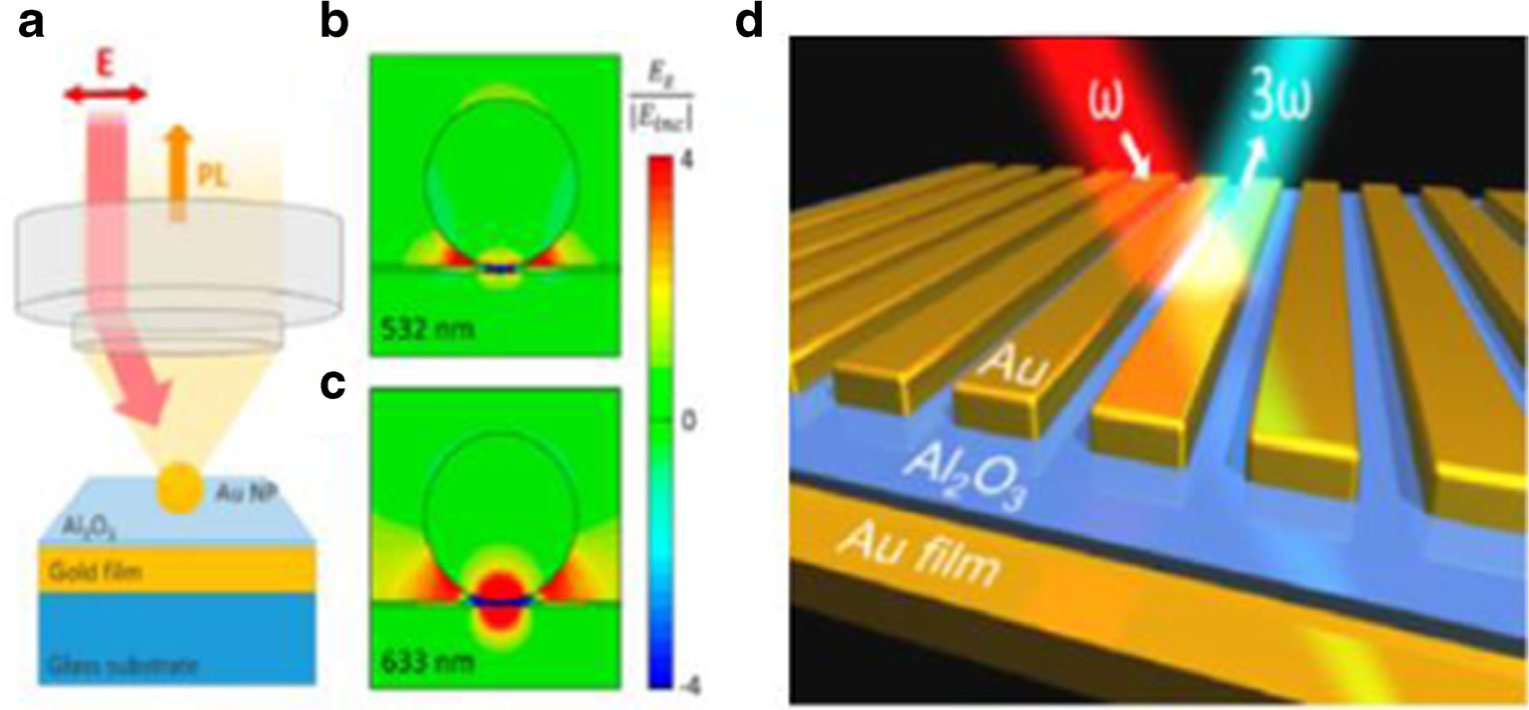
(**a**) Au nanoparticle over Au thin film separated by Al_2_O_3_ thin film.(**b**, **c**) The enhanced-local field over incident field with excitation at 532 and 633 nm. [71] (**d**) Au resonator over Au thin film separated by Al_2_O_3_ thin film [72]

Controlling of the spacer thickness of the metal nanoparticle over metallic thin film system has been also used to enhance the photoemission and third harmonic generation. Lumdee et al. [71] fabricated the Au nanoparticle over Au thin film system with an ultrathin Al_2_O_3_ spacer (3.4 nm) by ALD (Fig. 12a, b, c). The photoluminescence measurement showed this system could enhance the radiative recombination of the holes in the d-band with the electrons in the sp.-band much with both excitation wave of 532 and 633 nm. For the 633 nm excitation, an enhancement factor of 28,000 has been achieved, since the match of the excitation wave of Au and the plasmon resonance. Lassiter, et al. [72] fabricated an Au grating over Au thin film separated by an Al_2_O_3_ thin film deposited by ALD (Fig. 12d). The nanoscale junction between each strip formed a waveguide cavity resonator, and resulted in strong enhancement of electric field. The plasmon resonance could be changed by varying the strip width. The third harmonic generation enhancement depended on the spacer thickness strongly, which could increase to 10^5^ while the spacer thickness decreased to 2 nm.

**Fig. 13:**
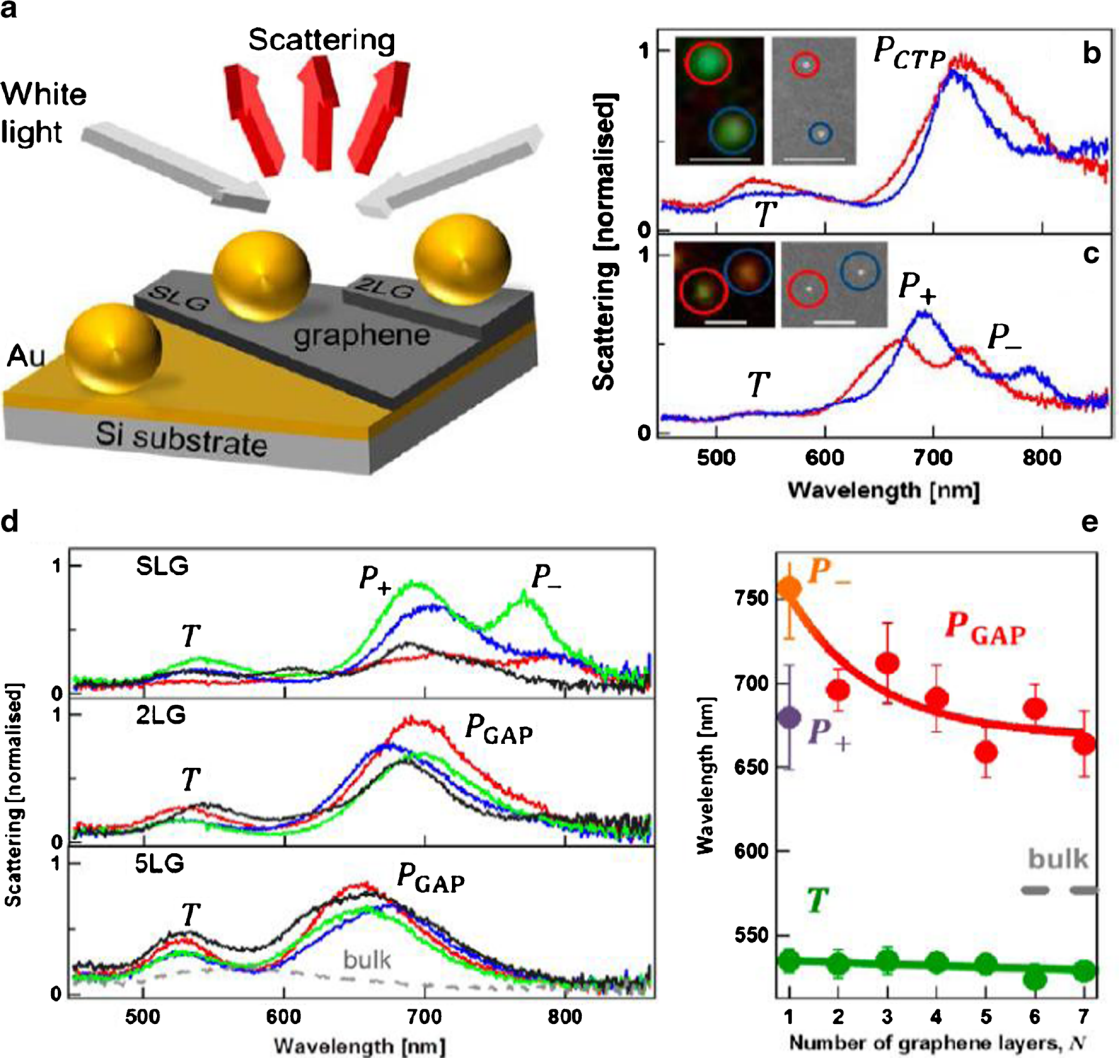
(**a**) Schematic of the gold nanoparticles/graphene/gold thin film model. (**b**) Dark field single particle scattering spectra for two gold nanoparticles directly on gold thin film.(**c**) Dark field single particle scattering spectra for two gold nanoparticles on monolayer graphene transferred onto gold thin film. (**d**) Dark field modes for “gold nanoparticles/graphene/gold thin film” model with SLG, 2LG, and 5LG spacer, respectively. (**e**) Resonance wavelength of the model depends on the number of graphene layers. [69]

As a nanoparticle dimer, when the spacer thickness is at subnanometer scale, the plasmonics would change from classical region to quantum region. Mertens et al. [68] successfully controlled the interception between gold nanoparticle and gold thin film to 0.34 nm using a graphene spacer. The single layer graphene (SLG) was first deposited on a Cu substrate with CVD, and then transferred to PMMA film via spinning PMMA on the surface of graphene and etching over all the Cu substrate. The remained PMMA/graphene film was then placed on a gold film with a thickness of 100 nm. PMMA was finally removed by acetone dissolution. The self-assembly gold nanoparticles with diameter of 80 nm was transferred to the surface of the graphene spacer using the same method as transferring graphene film. The schematic of the gold nanoparticles/ graphene/ gold thin film model is shown in Fig. 13a. While the gold nanoparticles sit on the gold thin film directly, two plasmon modes, the T mode and the charge-tunneling plasmon (CTP) mode, appeared at 530 and 720 nm, respectively. By contrast, while the gold nanoparticles were separated from the gold thin film with an SLG, two modes appeared at near-infrared region (P+/P-), but one mode (T mode) did not change. The scattering spectra of the model with SLG, 2LG, and 5LG as spacer, respectively, are shown in Fig. 13d, and the resonance wavelength depending on spacer thickness is shown in Fig. 13e. There was spectra doublet only for the one with SLG spacer, while the long wavelength mode blueshift with the increase of spacer thickness. The variation in modes in the near-infrared area with SLG as spacer has symbolized the hybrid plasmon started to go to quantum region from classical region based on Mertens et al.’s analysis [68].

The quantum effect in the coupling between LSP and SPP has been studied and verified by Kravtsov et al. [35] with a tip (emitter)-sample (gold) system with controllable distance. The measurement of PL and TERS have been performed to inspect the emission from the tip emitter with the distance down to subnanometer. The radiative emission was from the recombination of d-band holes and sp.-band electrons in gold. The 3D PL spectrum is shown in Fig. 13a. With the decrease of the tip-sample distance, the PL intensity increases because of the plasmon enhancement. While the tip-sample distance reduced to within 2 nm, strong quenching happened. The PL spectra actually can be identified as three parts: (1) with a long tip-sample distance, the far-field interference resulted in smooth increase of moderate intensity; (2) tip-sample distance further down to near-field region, the PL spectra redshift (z~5 nm); and (3) tip-sample distance down to less than 1.5 nm, a regime of quenching and significant line broadening appeared. The quantum effect can be seen in Fig. 13c, d. When there was no tunnel effect, the classical model showed that the PL intensity would increase with the decrease of the tip-sample distance (red dash line). However, considering the tunneling effect, the PL intensity would increase with the decrease of tip-sample distance, but it would decrease in subnanometer (red solid line). The experiment also showed that the PL intensity would decrease within subnanometer, which was in agreement with the quantum model (black circle). The TERS measurement used the C-H stretch vibrational mode of hydrocarbon as a probe between the tip and the gold film (Fig. 13d*).* Similar to the PL measurement, the increase in Raman intensity with the decrease of tip-sample distance for the non-tunnel mode (red dash line) was much higher than the quantum model (red solid line) within subnanometer. And the experiment data could match with the quantum model rather than the non-tunnel mode within subnanometer. In addition, (Fig. 13e) showed the schematic of the tip-sample model in classical (dipole-dipole coupling) and in quantum regime (charge transfer).

**Fig. 14:**
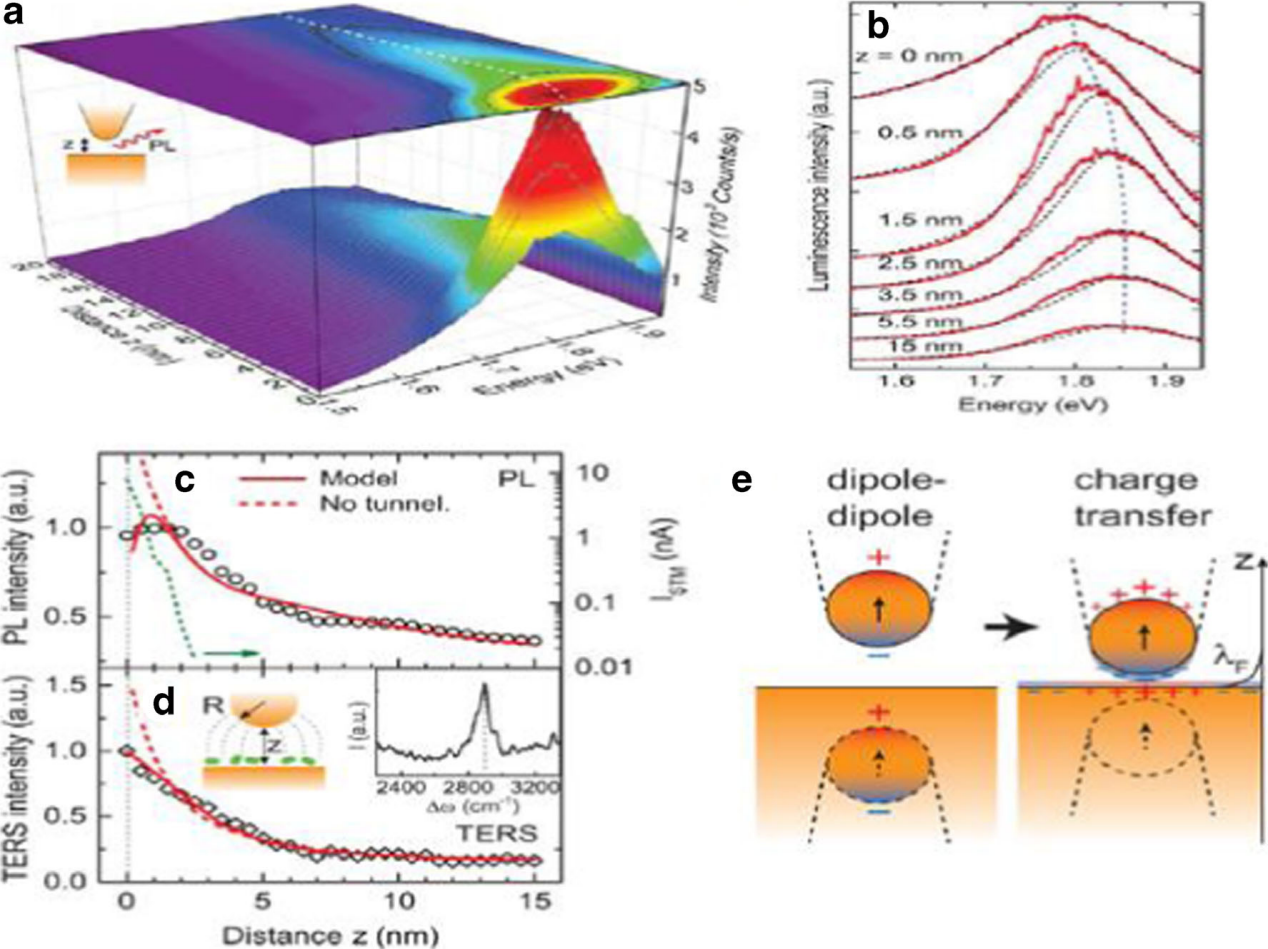
(**a**) 3D image for PL spectrum. (**b**) PL spectrum. (**c**, **d**) PL and TERS intensity depends on gold tip-gold film distance. (**e**) Classical and quantum coupling with different distance [35]

Furthermore, besides the LSP-SPP coupling model (either classical or quantum model), another similar model, LSP-(Micro) FP cavity has been studied in detail recently [19]. The nanostructure can take the advantage of a Fabry-Perot cavity, which could improve the remote-sensing ability of nanosensor (Fig. 14).

## Conclusion and Perspective

To summarize, the physics models of single nanoparticle, hybrid single nanoparticle, nanodimer, and the single nanoparticle closely over a metallic thin film, have been reviewed above. Theoretically, the models can be explained by classical electromagnetics theory and quantum theory. In nanoscale, the classical electrodynamics is enough to explain the near field, resonance spectrum of both the surface-propagating plasmonics and localized-surface plasmonics. But while the size of the nanoparticle down to less than 10 nm, the quasistatic assumption for the Maxwell’s equations would not work. Considering of the retardation effect, it needs to introduce the quantum theory for it. For the nanodimer with a junction in nanoscale, the classical eletrodynamic can also explain it successfully. But when the size of the junction down to subnanometer, the classical electrodynamics would fail to predict the resonance spectrum. The charge transfer or tunneling between two closely spaced nanoparticles should be considered, thus, both quantum corrected electromagnetics and quantum theory were used to explain it. For the single nanoparticle over the metallic thin film, the situation is quite similar to the nanodimers, because it can be also considered as a kind of coupling between different plasmonics: the localized plasmonics from the single nanoparticle and the surface-propagating plasmonics from the metallic thin film.

In fact, the models can be divided into two sorts. The single plasmonics and the hybrid plasmonics. The single nanoparticle produces single plasmonics [36]. For the hybrid single nanoparticle [22, 24, 25], it has been considered as the coupling between two different kinds of plasmonics. Especially, for the nanodimers [26] and the single nanoparticle closely over the metallic thin film [35, 69], the former is considered as the coupling between the localized-surface plasmonics from each nanoparticle in the nanodimer; the latter is considered as the coupling between the localized-surface plasmonics from the single nanoparticle and the surface-propagating plasmonics from the metallic thin film. Furthermore, these two models have broad applications in photonics and biophotonics [69, 71, 72].

The junction between the two nanoparticles in a nanodimmer, also named as hotspot, in which the highly-localized near field produced. In the surface-enhanced Raman scattering for ultralow concentration molecules detection, the molecule localized at the hotspot would contribute hugely to the total Raman signature’s intensity, which even can let single molecule detection possible. Normally, smaller the gap is, higher the localized field is, thus, achieve higher enhancement of the Raman signature’s intensity. Actually, the enhancement factor is defined as the ratio between the localized field and the incident field [9]

20 $$ \begin{array}{l}{\mathrm{EF}}_{\mathrm{SERS}}=\frac{{\left|{E}_{\mathrm{LOC}}\left({\omega}_L\right)\right|}^2{\left|{E}_{\mathrm{LOC}}\left({\omega}_R\right)\right|}^2}{{\left|{E}_{\mathrm{in}}\left({\omega}_L\right)\right|}^2{\left|{E}_{\mathrm{in}}\left({\omega}_R\right)\right|}^2}\\ {}\end{array} $$

Where (|*E*_in_(*ω*_*L*_)|^2^) is from the incident field of laser wave, (|*E*_in_(*ω*_*R*_)|^2^) represents the emission of Raman radiation, and (|*E*_LOC_(*ω*_*L*_)|^2^) represents the amplified local field of laser wave, (|*E*_LOC_(*ω*_*R*_)|^2^) represents the amplified local field of the Raman radiation.The nanofabrication of the SERS substrate was focused on design and fabricate high density, highly localized hotspots for magnification of the Raman scattering from molecules, no matter what kind of methods people used to produce the SERS substrates. The closely spaced silver nanodimers with an interception less than 10 nm by Wang et al. [73] showed high performance in detection of R6G in Raman microscopy. The synthesized-gold nanodimers can reach an even smaller nanogap as 3.3 nm [60], thus leads to high sensitivity test of DNA in Raman microscopy, even the influence of the laser polarization can be tested with this method [74]. With a wider sight of the nanodimers, closely spaced high density nanoparticles [75–78], or nanopillars [56, 79, 80], can be seen as multiple nanojunctions or hotspots. It has great application in SERS [56, 78, 79, 81]. The coupling between the-localized surface plasmonics and the surface-propagating plasmonics, can even enhance the Raman signatures and lead to high sensitivity detection [33, 70, 82]. Especially, when the different kinds of couplings combine together [33, 73]: the coupling between LSP and LSP, also the coupling between LSP and SPP, which would improve the SERS furtherly.

In the research and development, the theory models about plasmonics can be worked as a tool or a method for the designing of metallic nanostructures. Reversely, the nanofabrication can approve the theoretic models, or even complement the theoretic models, and also leads to practical applications, as well as, the practical applications can give feedback for further development of the nanostructure and fabrication.
